# Modification of Magnetite Nanoparticles with Triazine-Based Dendrons and Their Application as Drug-Transporting Systems

**DOI:** 10.3390/ijms222111353

**Published:** 2021-10-21

**Authors:** Mateusz Pawlaczyk, Grzegorz Schroeder

**Affiliations:** Faculty of Chemistry, Adam Mickiewicz University in Poznań, Uniwersytetu Poznańskiego 8, 61-614 Poznań, Poland; schroede@amu.edu.pl

**Keywords:** triazine-based dendrons, magnetite nanoparticles, magnetic hybrid materials, in vitro drug delivery, adsorption

## Abstract

The following research aims at the synthesis of magnetite nanoparticles functionalized with triazine-based dendrons and the application of the obtained materials as effective sorptive materials dedicated to acidic bioactive compounds. The adopted synthetic approach involved: (1) the synthesis of nanosized Fe_3_O_4_ particles via classic co-precipitation method, (2) the introduction of amine groups on their surface leading to materials’ precursor, and (3) the final synthesis of branched triazine-based dendrons on the support surface by an iterative reaction between cyanuric chloride (CC) and piperazine (p) or diethylenetriamine (DETA) via nucleophilic substitution. The characterized materials were tested for their adsorptive properties towards folic acid, 18β–glycyrrhetinic acid, and vancomycin, showing high adsorption capacities varying in the ranges of 53.33–401.61, 75.82–223.71, and 68.17–132.45 mg g^−1^, respectively. The formed material–drug complexes were also characterized for the drug-delivery potential, performed as in vitro release studies at pH 2.0 and 7.4, which mimics the physiological conditions. The release profiles showed that the proposed materials are able to deliver up to 95.2% of the drugs within 48 h, which makes them efficient candidates for further biomedical applications.

## 1. Introduction

Recently, hybrid materials based on oxide nanoparticles [1,2], especially magnetite (iron(II,III) oxide; Fe_3_O_4_) nanoparticles, gained significant attention as starting materials for the synthesis of tools dedicated to chemical analysis, adsorbents, supports for the delivery of various biocompounds, or catalysts, owing to their various beneficial physicochemical features. Namely, magnetite nanoparticles might be obtained using a wide range of easily accessible synthetic procedures (co-precipitation, thermal decomposition, hydrothermal method, etc.), which finally exhibit magnetic susceptibility, and thus might be easily separated using an external magnetic field. Moreover, due to the presence of multiple surface hydroxyl groups, magnetite particles are prone to direct surface functionalization or encapsulation within the silica matrix, leading to easily tunable Fe_3_O_4_/SiO_2_ systems [3,4,5,6,7].

The choice of functionalizing agents is always driven by the targeted applications of the designed materials. Among a plethora of organic domains used for surface functionalization, dendritic structures have gained significant attention due to their multifunctionality and biocompatibility, which influence a wide range of dendrimer-modified hybrid materials’ applications [8,9,10]. The family of dendrimers includes a type of triazine dendrimers, which contain triazine rings interconnected by a polynucleophilic agent, especially linear diamines or aminoalcohols. Characteristic features of triazine-based dendrimers are easiness in tunability afforded by chlorine atoms in the triazine-core undergoing nucleophilic substitution under mild conditions, the rigidity of the final structure afforded by the stiffness of triazine aromatic ring, and a combination of both hydrophobic and hydrophilic domains in the macromolecule. This kind of dendrimers was introduced to chemical nomenclature in the early 2000s [11] and was studied for the improvement of pharmacokinetic parameters of chosen drugs, with the simultaneous possibility of enhancing their therapeutic effects [12,13,14,15,16], delivering nucleic acids to cancer cells [17], as well as promising binding efficiency towards toxic metal ions [18,19].

Hence, the process of immobilization of triazine-based dendrimers on the inorganic support surface may lead to obtaining hybrid materials that have versatile applications since the dendritic receptors may form coordinating, electrostatic, π–π stacking, or inclusive interactions with analytes. Therefore, several materials containing surface triazine dendrimers or triazine-based dendrons have been investigated for the chelation of toxic metal ions, including Pb(II), Cu(II), or Hg(II) ions, as well as toxic organic compounds, such as dyes or phenol derivatives [20,21,22,23,24]. The metal binding properties also triggered the application of such materials as metal-containing catalysts dedicated to various organic reactions, affording excellent conversion rates and chemo- or stereoselectivity [25,26,27,28,29]. Surprisingly, hitherto literature presents only a few reports concerning the bioapplication of the materials functionalized with triazine-dendrimers. Nevertheless, magnetite nanoparticles conjugated with methotrexate through triazine dendrimers or dopamine–triazine linkers were investigated for enhanced drug delivery to various cell cultures [30,31].

In the context of the applicability of triazine-based dendrimers and dendrons in drug delivery and the insufficiency in current research studies, the following article concerns the synthesis of magnetite nanoparticles functionalized with triazine-dendrons and their further application as sorptive materials towards chosen bioactive compounds, which were folic acid, 18β–glycyrrhetinic acid, and vancomycin. The studies should address the characterization of the designed materials and investigate the influence of the structure of anchored triazine-based dendrons on the materials’ adsorptive properties, as well as their ability to transport the chosen drugs based on the drug release to aqueous media.

## 2. Results and Discussion

### 2.1. Synthesis and Characterization of Triazine Dendron-Functionalized Magnetite Nanoparticles

**Figure 1:**
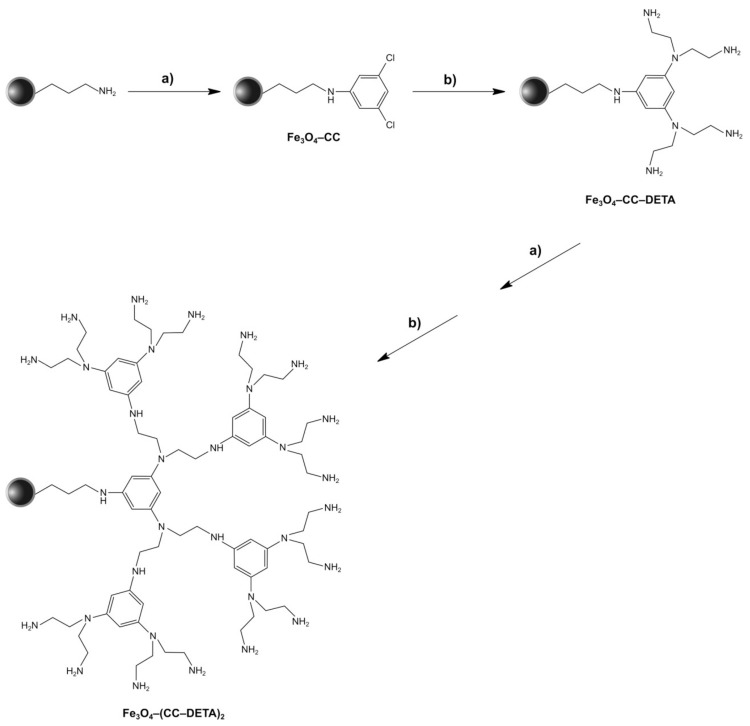
The synthetic protocol for obtaining the triazine dendron-functionalized magnetite nanoparticles as an introduction of cyanuric chloride (CC) and diethylenetriamine (DETA) to the surface of Fe_3_O_4_-NH_2_: (a) CC, THF, 2 °C—2 h; (b) DETA, ACN, RT—16 h, reflux—24 h.

**Figure 2:**
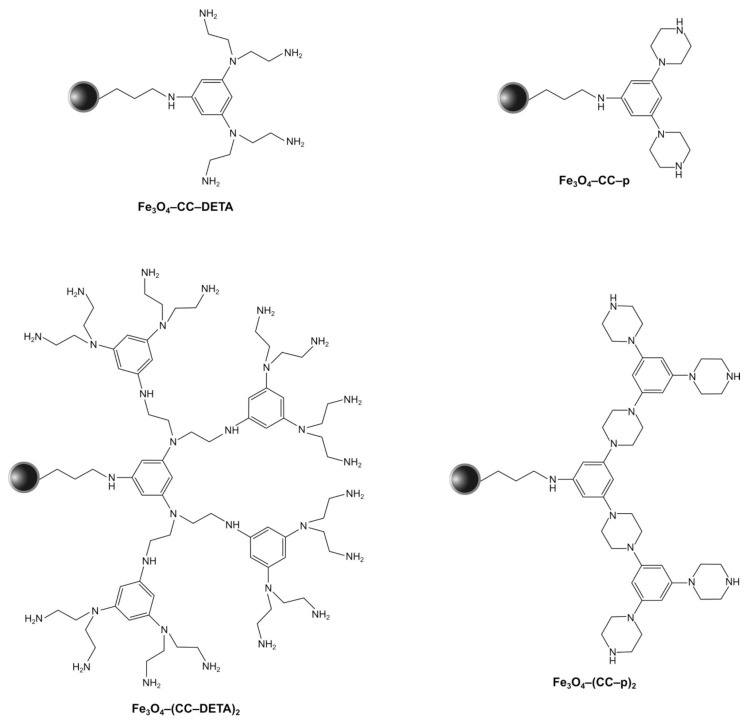
The structures of the obtained the triazine dendrons-modified magnetic nanoparticles.

The nanoparticles of magnetite were synthesized following the standard co-precipitation approach, while their further encapsulation with a thin silica layer was performed via the Stöber process [32]. The prepared Fe_3_O_4_/SiO_2_ particles were then subjected to functionalization with a silane derivative containing aminopropyl chain, leading to a magnetically susceptible material with free terminal NH_2_ groups as a precursor for the synthesis of the designed triazine dendron-grafted materials. Obtaining the final hybrid materials was based on two steps: (a) the incorporation of cyanuric chloride (CC)—a branching unit—to terminal amine groups and (b) the introduction of the aminocomponent (diethylenetriamine (DETA); or piperazine (p)). The aforementioned synthetic approach is illustrated in Figure 1. Each of the steps is based on the substitution of chlorine atoms of cyanuric chloride with amine groups, either anchored to the surface or added as a reactant. The main advantage of the adopted method is the chemoselectivity of chlorine atoms undergoing nucleophilic substitution at different reaction conditions. Namely, the substitution of the first chlorine atom takes place at a lowered temperature of <2 °C, the second atom at room temperature, and the third at elevated temperature (reflux); thus, the temperature control allows for easy directing of the dendrons growth. Therefore, following the described synthetic protocol, two materials containing first-generation dendrons, G1 (only one branching level; materials Fe_3_O_4_–CC–p and Fe_3_O_4_–CC–DETA), and two materials containing second-generation dendrons, G2 (two branching levels; materials Fe_3_O_4_–(CC–p)_2_ and Fe_3_O_4_–(CC–DETA)_2_), were obtained, which are presented in Figure 2.

**Figure 3:**
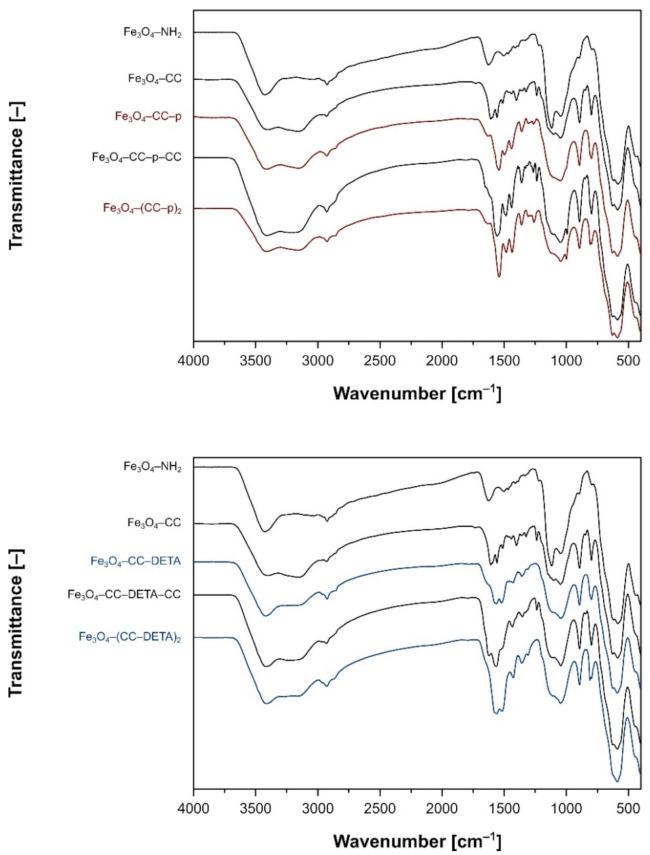
The FT–IR spectra of the magnetic support (Fe_3_O_4_–NH_2_), the cyanuric chloride (CC) intermediates (Fe_3_O_4_–CC and Fe_3_O_4_–CC–amine–CC), and the dendron-functionalized materials (Fe_3_O_4_–CC–amine and Fe_3_O_4_–(CC–amine)_2_) containing piperazine (p) (top) and diethylenetriamine (DETA) (bottom) as an amine domain.

All the synthesized materials and their intermediates were characterized with FT–IR analysis, and the spectra are presented in Figure 3. Each spectrum exhibits several bands, common for all the materials, including a very strong absorption band of Fe–O lattice stretching centered approximately at 590 cm^−1^, a broad band with a maximum at around 1050 cm^−1^ assigned for the stretching vibrations of Si–O–Si domains, a relatively low intense signal of –CH_2_– stretching at around 2925 cm^−1^, or a very broad band between 3100 and 3500 cm^−1^ with a maximum at around 3410 cm^−1^, corresponding to either the stretching of free hydroxyl groups on Fe_3_O_4_/SiO_2_ surface or/and the stretching of N–H domains. Additionally, after the first step of incorporation of cyanuric chloride to the nanomaterial’s surface, new, common for all the functionalized materials, signals at 798, 894, and broad signal 1600 and 1660 cm^−1^ appeared, referring to the vibrations of the trisubstituted aromatic ring of triazine. Moreover, with the growth of triazine-based dendrons on the materials’ surface, several signals between 1300 and 1500 cm^−1^, corresponding to various C–N stretching, or C–H and N–H bending, became more intensive, which proved the successful grafting the reagents on the materials. Interestingly, the steps of cyanuric chloride incorporation were undoubtedly proved by the appearance of signals at around 843 and 1238 cm^−1^, which are related to the vibrations of C–Cl.

All the dendron-functionalized materials were tested for the content of the terminal amine groups based on the classic acid–base titration method. The calculated loading of amine groups *L_a_* and loading of the dendrons’ residues *L_d_* are presented in Table 1. The loading values of amine groups calculated for the materials containing piperazine as the aminocomponent are noticeably lower than the corresponding values for DETA-containing materials, which is related to the structural features of both amines used. However, the values of quantified dendrons’ loading are significantly higher for piperazine-based dendrons than those calculated for DETA-based dendrons. Such results are strictly connected with the various reactivity of structurally different amines for the substitution of chlorine atoms in cyanuric chlorine, which are mainly attributed to steric effects and amine’s pK_a_ values, and can be presented in the following order: cyclic aliphatic amines > linear aliphatic amines > aryl amines [33]. Therefore, the first and the second incorporation of piperazine domain to the materials Fe_3_O_4_–CC–p and Fe_3_O_4_–(CC–p)_2_, respectively, was achieved with a higher yield and with insignificant yield loss during the second substitution step, unlike the incorporation of DETA domains into the corresponding materials.

**Figure 4:**
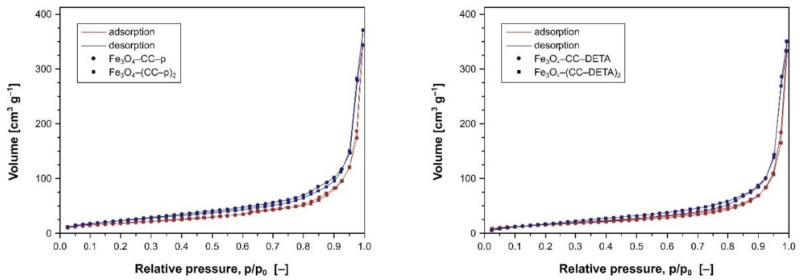
The BET isotherms of the materials containing piperazine-dendrons (**left**) and DETA-dendrons (**right**).

Moreover, the materials containing full dendrons of the first or second generation were analyzed for their porous features using the N_2_ adsorption/desorption method, which isotherms are presented in Figure 4, while the obtained porosity parameters are listed in Table 1. The effect of the dendrons’ generation on the porous properties of the materials is rather insignificant, which is proved by a high similarity of the corresponding isotherms obtained for the materials grafted with dendrons of the first and second generation. The pore size values calculated for the materials containing first-generation piperazine-dendrons are lower than those calculated for DETA-dendrons, which is connected with the branching character of both amines. Piperazine as the structurally compact amine creates the smaller cavities, while DETA triggers the creation of bigger pores since its attachment leads to branched units. On the other hand, the second coupling step promotes the formation of a bigger size for Fe_3_O_4_–(CC–p)_2_, while smaller for Fe_3_O_4_–(CC–DETA)_2_, which is connected with loosing and tightening of the dendrons, respectively. These findings are consistent with pore volume values, also listed in Table 1.

### 2.2. Investigation of Adsorptive Properties of the Dendron-Functionalized Magnetic Materials towards Bioactive Compounds

**Figure 5:**
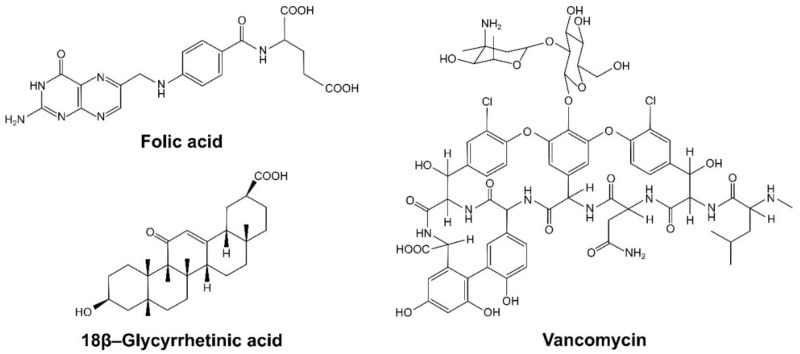
The structures of the biocompounds used in the studies.

The synthesized and characterized materials have been studied for their binding potential of three biocompounds: folic acid, 18β–glycyrrhetinic acid, and vancomycin, which are presented in Figure 5. The choice of these biologically active agents was predominantly driven by their acidic character (presence of free carboxylic groups), which triggers their attractive interaction through the exchange of a proton between the –COOH domain of the analytes and free terminal –NH– or –NH_2_ groups of the synthesized triazine-based dendrons. Such an exchange leads to a formation of two oppositely charged species undergoing electrostatic interaction, and thus the drugs adsorb on the materials’ surface.

**Figure 6:**
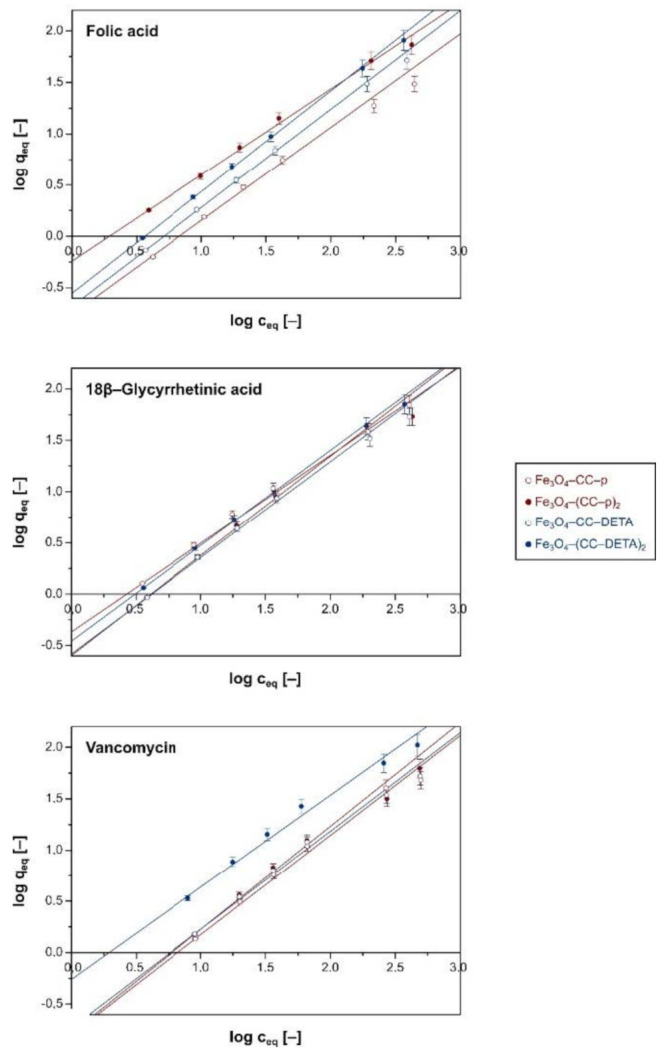
The fitting of the experimental data of the adsorption of the biomolecules on the surface of the dendron-functionalized hybrid materials by the Freundlich model.

All the obtained dendron-modified supports were subjected to adsorption isothermal studies. The experimental data were further fitted to the Langmuir (Supplementary Figure S1) and the Freundlich (Figure 6) models, which parameters are collected in Table 2. Each of the adsorption processes followed the Freundlich isothermal model, proved by significantly higher linear correlation coefficient R^2^ values than those calculated for the Langmuir model, as well as lower values of χ^2^, proving better correlation with the regression line. Such results are easily explainable since the Freundlich model assumes the probability of the intermolecular interactions of adsorbate molecules, which potentially happens through the π–π stacking effect of phenyl rings of the analytes. The calculated 1/*n* parameters, connected with the strength of the adsorption process, reached very high values >0.80, indicating the high adsorptive potential of the materials [34,35]. Nevertheless, on the basis of the experimental data fitting to the Langmuir model, the maximal adsorption capacity values *q_m_* were calculated, which are collected in Table 2. The adsorptive potential of the materials towards the chosen drugs is strictly dependent on both the structure of the pending triazine-based dendrons and the structure of the drugs. The materials containing double-branched dendrons ((CC–p)_2_ and (CC–DETA)_2_ domains) showed two times higher adsorption capacities than the materials containing corresponding single-branched domains, which is caused by the presence of a higher number of terminal amine groups that are able to interact with the drugs molecules. Additionally, the use of DETA as an aminocomponent has led to the materials of higher sorptive potential than those containing piperazine as a repetitive block, which is due to the (a) better branching character of DETA, leading to more expanded dendritic structures, and (b) the incorporation of a higher number of free –NH_2_ groups on the materials’ surface. Taking the aforementioned dependence into account, the least adsorptive material is material Fe_3_O_4_–CC–p, in which the adsorption capacities towards the biomolecules varied between 53.33 and 75.82 mg g^−1^, while the most potent adsorptive material is Fe_3_O_4_–(CC–DETA)_2_, in which the sorptive capacity was in the range of 132.42–401.61 mg g^−1^ for the drugs used. On the other hand, the structure of the adsorbate’s molecules also influences their ability to interact with the surface of the materials. Among the three used drugs, folic acid is the only molecule containing two carboxylic molecules, leading to an enhanced stabilization of its electrostatic interactions with amine groups on the materials’ surface, which is especially proven for its adsorption on the materials functionalized with DETA-containing dendrons, reaching *q_m_* values of 170.07 and 401.61 mg g^−1^ for Fe_3_O_4_–CC–DETA and Fe_3_O_4_–(CC–DETA)_2_, respectively. In the case of vancomycin—a glycosylated polypeptide—the carboxylic group is hindered with a sterically expanded residue, which might decrease its ability to interact with the materials. Nevertheless, the materials showed an adsorption capacity towards vancomycin in the range of 68.17–132.45 mg g^−1^.

**Figure 7:**
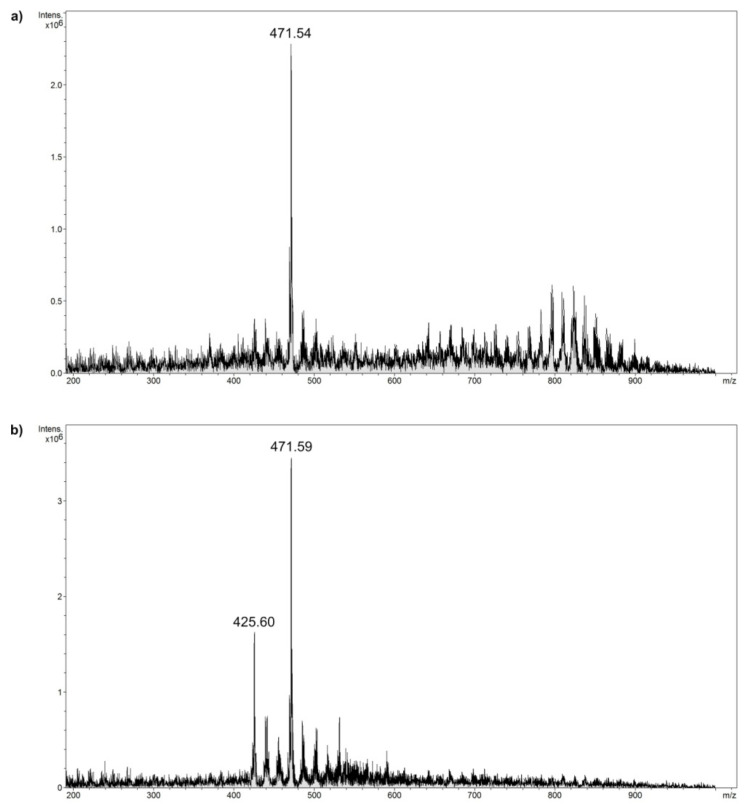
The FAPA–MS spectra of 18β–glycyrrhetinic acid thermally desorbed from its complexes with: (**a**) Fe_3_O_4_–(CC–p)_2_ and (**b**) Fe_3_O_4_–(CC–DETA)_2_ hybrid materials.

The materials containing the dendrons of the second generation ((CC–p)_2_ and (CC–DETA)_2_ domains) with the adsorbed drugs on their surface were subjected to FAPA–MS analysis, which involves the ionization of the analyte in the flow of plasma generated outside the spectrometer using the Flowing Atmospheric-Pressure Afterglow (FAPA) ionization technique. In the case of the three studied biocompounds, the only one which was ionizable from the material–drug complexes in the plasma stream was 18β–glycyrrhetinic acid. The FAPA–MS spectra of materials Fe_3_O_4_–(CC–p)_2_ and Fe_3_O_4_–(CC–DETA)_2_ with adsorbed 18β–glycyrrhetinic acid are presented in Figure 7, and the results undoubtedly prove its successful adsorption onto the surface of triazine-based dendron–functionalized materials by the appearance of a signal at *m*/*z* 471.5 related to the drug’s molecular peak. Additionally, the spectrum of Fe_3_O_4_–(CC–DETA)_2_ exhibits an additional intensive signal at *m*/*z* 425.6, which corresponds to a fragmentation signal of 18β–glycyrrhetinic acid after a loss of carboxylic group. This signal was observed as the main fragmentation signal in the MS^2^ spectrum during fragmentation of molecular peak *m*/*z* 471.5 of 18β–glycyrrhetinic acid.

### 2.3. Investigation of the Materials’ Drug-Delivery Potential

The delivery potential of the materials functionalized with triazine-based dendrons of the second generation (materials Fe_3_O_4_–(CC–p)_2_ and Fe_3_O_4_–(CC–DETA)_2_) towards the studied three drugs was investigated by the monitoring of the drugs’ release from their material–drug complexes in two different aqueous media: HCl/KCl buffer of pH 2.0 and phosphate-buffered saline (PBS) of pH 7.4. The choice of such release media was driven by the conditions, which mimic the conditions of human fluids: gastric juice and human plasma, respectively.

**Figure 8:**
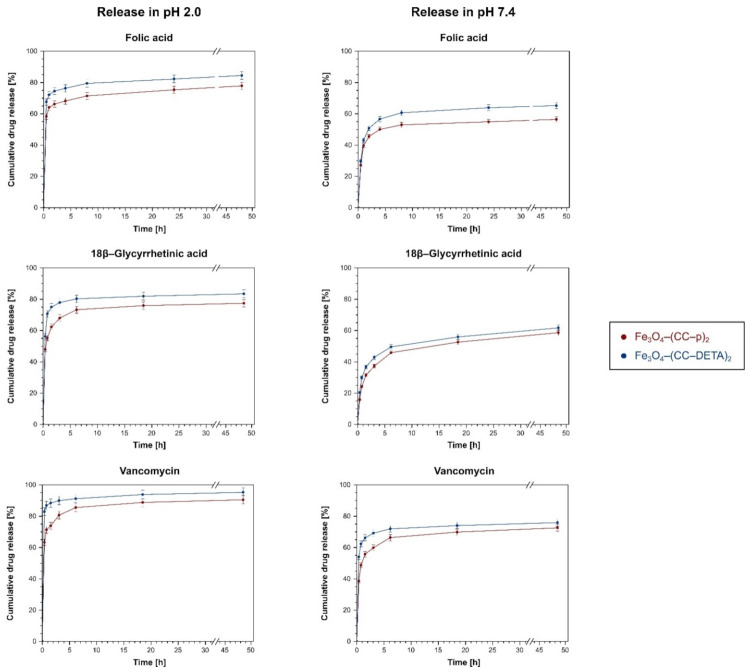
The profiles of the drugs releases from their complexes with Fe_3_O_4_–(CC–p)_2_ (red) and Fe_3_O_4_–(CC–DETA)_2_ (blue) performed in two different environments: pH 2.0 (**left**) and pH 7.4 (**right**).

The investigated release profiles (Figure 8) show that in an acidic environment of pH 2.0, a burst release appears within the first 2 h, which is connected with the drastic protonation of drugs’ carboxylate domains, hindering the attractive interactions between the materials and the drugs. Such a burst release led to the release of approximately 65–85% of the drugs in 2 h, with a slight drug release increase within the next 2 days of incubation. On the other hand, the experiments involving the material–drug complexes’ incubation in PBS solution of pH 7.4 showed that the progressive release of the biomolecules reached a quasi-plateau after 8 h for folic acid and vancomycin. In the case of 18β–glycyrrhetinic acid, its release in pH 7.4 proceeded with a continuous manner within the full time of incubation. Moreover, in each release profile, a similar trend of a slightly higher release percentage from Fe_3_O_4_–(CC–DETA)_2_ than Fe_3_O_4_–(CC–p)_2_ is observed. It may be caused by the more disruptive influence of the adsorbing agents used towards interactions between carboxylate groups of the drugs and protonated –NH_3_^+^ groups of the terminal DETA domain than those of the –NH_2_^+^– groups of piperazine. Furthermore, among all the studied drugs, vancomycin exhibits its highest release percentage from the triazine-based dendron-functionalized materials, varying between 72.5% and 95.2%, which might be a result of the least intensive interaction between vancomycin and the materials’ surface due to the drug’s steric hindrance. Nevertheless, the cumulative drug release after 48 h incubation of folic acid– and 18β–glycyrrhetinic acid-loaded materials ranged between 56.4% and 84.5% and 58.6% and 83.4%, respectively, indicating their efficient release to the aqueous media.

The experimental data of the drug releases from the hybrid materials were also fitted to several drug release kinetic models, which were the zero-order, the first-order, the Higuchi, the Hixson–Crowell, and the Korsmeyer–Peppas models, which linear forms are collected in Table 3. Among all the proposed models, the Korsmeyer–Peppas model showed the best fitting to the investigated release experiments, which is proved by the highest values of R^2^ correlation coefficients (Table 4). Accordingly, it can be postulated that the drugs’ release from the hybrid materials follows the Fickian or quasi-Fickian diffusion model, which is connected with the calculated *n* values significantly lower than 0.45 [36]. The fitting of experimental data to the zero-order, the first-order, and the Hixson–Crowell models gave very low R^2^ values (Supplementary Table S1), which indicates that the drugs’ releases do not follow these models.

## 3. Materials and Methods

### 3.1. Chemicals

All the reagents used for the synthesis of triazine-dendrons, i.e., (3–Aminopropyl)– trimethoxysilane (97%), cyanuric chloride (cc) (99%), piperazine (p) (99%), diethylenetriamine (DETA) (99%), and diisopropylethylamine (≥99%), and the chosen analytes: vancomycin hydrochloride hydrate, 18β–glycyrrhetinic acid (97%), and folic acid (≥97%) were purchased from Sigma-Aldrich (Saint Louis, MO, USA). All the solvents and chemicals were of purity grade p.a. and were purchased from Stanlab (Lublin, Poland) (Hydrochloric acid conc., Sodium hydroxide, and Toluene), POCH (Gliwice, Poland) (Disodium phosphate dihydrate, Sodium phosphate monohydrate, and Potassium chloride), Eurochem BGD (Tarnów, Poland) (Sodium chloride, Tetrahydrofuran, and Dichloromethane), and Merck (Darmstadt, Germany) (Acetonitrile).

### 3.2. Instruments

The synthesized Fe_3_O_4_-based materials functionalized with triazine-based dendrons were characterized with transmission FT-IR spectroscopy using the Bruker IFS 66v/S (Bremen, Germany) spectroscope. The measurement conditions were as follows: wavelength range of 400–4000 cm^−1^; resolution of 2 cm^−1^; suspension of 1.5 mg of the sample in KBr pellet. The materials were also characterized with nitrogen adsorption/desorption isotherms (Brauner–Emmet–Teller; BET) using the Quantachrome Autosorb iQ (Boynton Beach, USA) analyzer. The adsorption conditions were as follows: sample outgassing for 12 h at 100 °C before the analysis; the temperature-controlled analyses were performed at 77.35 K; the time of analyses varied between 12.01 and 18.49 h; the measurements were handled in the range of relative pressure p/p_0_ between 0.0 and 1.0. The raw data were further analyzed in order to calculate porosity parameters of the analyzed materials using BET and Barrett–Joyner–Halenda (BJH) methods, applied in the range of p/p_0_ between 0.05 to 0.3 and 0.1 to 1.0, respectively.

The drugs’ adsorption progress and their release to the adsorbing media were monitored with UV-Vis measurements using the Agilent 8453 spectrophotometer (Santa Clara, USA). The liquid samples were placed in a poly(methyl methacrylate) (PMMA) cuvette of an optical path length of 10 mm. The spectra recorded in the wavelength range between 200 and 1000 nm, with a resolution of 1 nm, were conducted in triplicate to avoid any absorbance disturbances. The FAPA–MS spectra of the material–drug complexes were recorded using the Bruker amaZon SL ion trap (Bremen, Germany) spectrometer equipped with an L-FAPA ambient plasma source supplied by ERTEC (Wroclaw, Poland) [37]. External helium plasma as an ion source was generated by the gas flowing through a quartz tube at a rate of 1 L min^−1^, which gets discharged at the end of the tube by the open-circuit voltage of 20 kV, allowing the self-ignition of the gas. In order to analyze samples using such ionization mode, the samples were on mini-crucibles located approximately 5 cm below the plasma stream, which might have been heated up to 350 °C. The vertical distance between the FAPA ionization source and the MS ions inlet was approximately 10 cm. The mass spectrometer worked in a so-called ‘enhanced resolution mode’, affording the detection of *m*/*z* in the range of 50 to 2200 with a scanning rate of 8100 *m*/*z* per second. The spectrometer used helium as the cone gas at its flow rate of 50 L h^−1^.

### 3.3. Synthesis of Magnetite Nanoparticles Functionalized with Triazine-Based Dendrons

#### 3.3.1. Synthesis of Amino-Functionalized Magnetic Particles

The synthesis of magnetite nanoparticles encapsulated with silica matrix was performed according to the previously described procedure [38]. The obtained magnetically susceptible support was further functionalized with (3–aminopropyl)-tri- methoxysilane (APTMS) in order to introduce free terminal amine groups on the surface. Therefore, a solution of APTMS (392.8 μL; 2.25 mmol) in 50 mL of toluene was added dropwise into a suspension of 3 g of Fe_3_O_4_–SiO_2_ in 100 mL of toluene heated to a temperature of 50 °C with continuous stirring in an ultrasound bath. The sonification at a temperature of 50 °C was maintained for 5 h, and then the mixture was shaken at room temperature overnight. The resulting solid of Fe_3_O_4_–NH_2_ was magnetically separated, washed with toluene (2 × 50 mL) and DCM (2 × 50 mL), and dried under vacuum in a desiccator at room temperature.

#### 3.3.2. Quantification of the Amino Groups Loading

The amount of the amine groups anchored to the Fe_3_O_4_–NH_2_ surface was examined using the classic acid–base titration technique. Namely, a 20 mg sample of the material was mixed with 20 mL of 0.01 M aqueous solution of HCl at room temperature for 2 h. Afterwards, the solid was separated using a magnet, and the solute containing an excess of acid was titrated with 0.005 M aqueous solution of NaOH, using phenolphthalein as an indicator. The procedure was performed three times, calculating a mean value of amine groups’ loading.

#### 3.3.3. Synthesis of Fe_3_O_4_–CC Precursor

In the next step, cyanuric chloride (CC) was immobilized on the surface of the magnetite-based material. Therefore, to a solution of a 2-fold excess of CC (494.6 mg; 2.68 mmol) and DIPEA (467.2 μL; 2.68 mmol) in 150 mL of THF cooled to temperature ~2 °C in an ice-water bath, 3 g of Fe_3_O_4_–NH_2_ was added in several portions under continuous mechanical stirring, not to let the temperature rise. After the material’s addition, the mixture was stirred for 2 h at lowered temperature and then separated using a magnet. The solid was washed with THF (2 × 50 mL) and DCM (2 × 50 mL) and dried under vacuum at room temperature, yielding Fe_3_O_4_–CC in dark brown.

#### 3.3.4. Synthesis of Triazine-Dendrons Containing Piperazine

A solution of 1.5-fold excess of piperazine (173.3 mg; 2.01 mmol) and DIPEA (350.4 μL; 2.01 mmol) in 100 mL of ACN was purged N_2_, and then 1.5 g of Fe_3_O_4_–CC was added to the solution in a few portions under mechanical stirring at room temperature. The stirring was maintained for 16 h, and then the mixture was refluxed for another 24 h. Afterwards, the mixture was cooled, the solid was separated with a magnet, washed with ACN (2 × 30 mL) and DCM (2 × 30 mL), and dried in the desiccator at room temperature, yielding Fe_3_O_4_–CC–p in dark brown. Half of the obtained material (0.75 g) was further reacted again with cyanuric chloride (185.4 mg; 1.01 mmol) and piperazine (173.3 mg; 2.01 mmol) in the presence of DIPEA, following the same synthetic procedure of the substitution of CC (described in Section 3.3.3) and the subsequent substitution of piperazine (described above), obtaining a dark brown solid of Fe_3_O_4_–(CC–p)_2_.

#### 3.3.5. Synthesis of Triazine-Dendrons Containing Diethylenetriamine (DETA)

The adopted synthetic procedure for obtaining the dendrons containing cyanuric chloride and diethylenetriamine was similar to the one described in Section 3.3.4. Briefly, 1.5 g of Fe_3_O_4_–CC was added to an anhydrous solution of DETA (217.1 μL; 2.01 mmol) and DIPEA (350.4 μL; 2.01 mmol) in several portions and then mixed for 16 h at room temperature and for 24 h at reflux. Then, a half (0.75 g) of separated, washed, and dried Fe_3_O_4_–CC–DETA was added in a few portions to a cooled solution of CC (370.7 mg; 2.01 mmol) and DIPEA (350.4 μL; 2.01 mmol) at a temperature 2 °C in 75 mL of THF. The mixing was maintained for 2 h at a lowered temperature. Afterwards, the separated, washed, and dried material was added to a solution of DETA (434.3 μL; 4.02 mmol) and DIPEA (700.8 μL; 4.02 mmol) in 75 mL of ACN previously purged with N_2_. The mechanical stirring was continued for 16 h, and then the mixture was refluxed for 24 h, obtaining Fe_3_O_4_–(CC–DETA)_2_.

#### 3.3.6. Quantification of the Dendrons Loading on the Materials’ Surface

The procedure for the quantification of the amount of the amine groups introduced on the hybrid materials’ surface was the same as described in paragraph Section 3.3.2. The classic acid–base titration using 0.01 M aqueous solution of HCl and 0.005 M aqueous solution of NaOH was performed for all the materials containing surface triazine-based dendrons.

### 3.4. Sorption Experiments

#### 3.4.1. Adsorption of the Chosen Acidic Bioactive Compounds

The isothermal adsorption studies involved the incubation of 10 mg samples of Fe_3_O_4_–CC–p, Fe_3_O_4_–(CC–p)_2_, Fe_3_O_4_–CC–DETA, or Fe_3_O_4_–(CC–DETA)_2_ in a series of 10 mL biocompounds’ solutions at concentrations of 0.01, 0.025, 0.05, 0.1, 0.5, and 1 mM. The solvents were chosen according to the biocompounds used, i.e., distilled water for vancomycin, phosphate buffer pH 8.0 for folic acid, and distilled water:ethanol (1:3) mixture for 18β–glycyrrhetinic acid. The mixtures of the materials and the solutions were shaken for 24 h at room temperature. Afterwards, the materials were separated from the samples by centrifugation prior to separation using a magnet. The solutes were further analyzed using UV-Vis spectrophotometric assays, detecting the absorbance values at λ of 358, 254, and 280 nm for folic acid, vancomycin, and 18β–glycyrrhetinic acid, respectively. On the basis of the calculated concentration of the biocompounds remaining in the solution after the adsorption experiments, the amount of the drugs bound to the materials (*q_eq_*) was calculated using Equation (1):

(1) $$q_{eq}=\frac{\left(c_{0}-c_{eq}\right)\cdot V\cdot M}{m}$$

where *c*_0_ and *c_eq_* are the initial and equilibrium concentration of the biocompounds, respectively, (mM); *m* is the sample mass (mg); *V* is the volume of the solution (mL); *M* is the molar mass of the analytes (g mol^−1^). Moreover, the solids of Fe_3_O_4_–(CC–p)_2_ and Fe_3_O_4_–(CC–DETA)_2_ incubated with the most concentrated solutions of the biomolecules were also further dried under vacuum and subjected to FAPA–MS analysis.

#### 3.4.2. Biocompounds Release Experiments

Investigation of drug-delivery potential of the obtained materials was performed in two steps: (1) loading the drugs onto the surface of Fe_3_O_4_–(CC–p)_2_ and Fe_3_O_4_–(CC–DETA)_2_; (2) incubation of material–drug complexes in phosphate-buffered saline (PBS) or hydrochloric acid/potassium chloride buffer pH 2.0. Therefore, 50 mg samples of Fe_3_O_4_–(CC–p)_2_ and Fe_3_O_4_–(CC–DETA)_2_ were shaken with 20 mL of 1 mM solutions of vancomycin, 18β–glycyrrhetinic acid, or folic acid for 24 h at room temperature. Afterwards, the materials were centrifuged and separated using a magnet and then dried in the desiccator at room temperature. The amount of the drugs complexed on the materials’ surface was determined using UV-Vis measurements, as described in Section 3.4.1. Then, samples of the material–drug complexes (ca. 15 mg) were incubated in 5 mL of pre-prepared PBS or HCl/KCl buffer at room temperature. The buffer aliquots were replaced with a fresh buffer after 0.5, 1, 2, 4, 8, 24, and 48 h of incubation. The solutes were subjected to UV-Vis analysis in order to quantify the cumulative drug release *F_t_* and cumulative percentage of the drug released *Q_t_*, according to Equations (2) and (3), respectively:

(2) $$F_{t}=\overset{t}{\underset{i=0}{\sum }}c_{t}\cdot V\cdot M$$

(3) $$Q_{t}=\frac{F_{t}}{m_{0}}\cdot 100\%$$

where *c_t_* is the concentration of the released drug (M); *V* is the volume of the releasing medium (mL); *M* is the drug’s molar mass (g mol^−1^); *m*_0_ is the mass of the drug in the studied material–drug complex (mg).

## 4. Conclusions

Recent studies have shown the synthetic approach for obtaining the triazine-based dendron-functionalized magnetite nanoparticles, which were characterized for adsorptive properties towards the chosen acidic bioactive molecules. The iterative nucleophilic substitution steps led to obtaining branched domains on the support’s surface with a dendron-loading efficiency of 0.195, 0.180, 0.117, and 0.069 mmol g^−1^ for Fe_3_O_4_–CC–p, Fe_3_O_4_–(CC–p)_2_, Fe_3_O_4_–CC–DETA, and Fe_3_O_4_–(CC–DETA)_2_, respectively. The materials showed satisfactory adsorption properties towards folic acid, 18β–glycyrrhetinic acid, and vancomycin, depending on either the dendron’s structure or the adsorbate structure. Additionally, the materials containing second-generation dendrons exhibited promising in vitro drug-transport efficiency, described by a drug release percentage between 56.4% and 95.2% after incubation in paraphysiological buffered solutions. Therefore, such materials may be utilized as direct drug-delivery platforms, which can be easily directed by an external magnetic field. This physical property of the materials allows for their exact introduction to organs or tissues. Moreover, since the materials showed efficient binding of folic acid—a structure that biodirects to over-expressed folic acid receptors of mutated cells—such complex might be useful as an easily recognized agent for hyperthermia treatment. The mentioned bioapplications of the proposed materials, however, must be proved by proper assays and experiments, which are the nearest plans of our studies.

## Figures and Tables

**Table 1:** The amine groups loading and the porous properties of the final triazine dendron-functionalized materials.

Material	Loading (mmol g^−1^)		N_2_ Adsorption/Desorption Analysis		
	L_a_	L_d_	Surface Area (m^2^ g^−1^)	Pore Size (nm)	Pore Volume (cm^3^ g^−1^)
Fe_3_O_4_–CC–p	0.390	0.195	68.914	30.9	0.533
Fe_3_O_4_–(CC–p)_2_	0.719	0.180	71.672	32.1	0.575
Fe_3_O_4_–CC–DETA	0.467	0.117	58.259	86.0	0.542
Fe_3_O_4_–(CC–DETA)_2_	1.104	0.069	62.063	65.9	0.515

**Table 2:** The isothermal parameters calculated for experimental data of the drug adsorption on the synthesized materials functionalized with triazine-based dendrons.

Material	Langmuir Isotherm			Freundlich Isotherm		
	q_m_ (mg g^−1^)	R^2^	χ^2^	1/*n* (–)	R^2^	χ^2^
**Folic Acid**						
Fe_3_O_4_–CC–p	53.33 ± 4.44	0.9794	0.072	0.91 ± 0.02	0.9930	0.029
Fe_3_O_4_–(CC–p)_2_	123.92 ± 9.54	0.9476	0.044	0.84 ± 0.03	0.9971	0.008
Fe_3_O_4_–CC–DETA	170.07 ± 18.32	0.9880	0.047	0.96 ± 0.02	0.9991	0.005
Fe_3_O_4_–(CC–DETA)_2_	401.61 ± 10.56	0.9960	0.005	0.99 ± 0.02	0.9995	0.003
**18β–Glycyrrhetinic Acid**						
Fe_3_O_4_–CC–p	75.82 ± 4.90	0.9658	0.102	0.86 ± 0.01	0.9902	0.034
Fe_3_O_4_–(CC–p)_2_	176.99 ± 20.68	0.9801	0.009	0.97 ± 0.02	0.9987	0.007
Fe_3_O_4_–CC–DETA	114.16 ± 11.75	0.9894	0.019	0.94 ± 0.02	0.9969	0.015
Fe_3_O_4_–(CC–DETA)_2_	223.71 ± 21.68	0.9723	0.013	0.93 ± 0.02	0.9988	0.005
**Vancomycin**						
Fe_3_O_4_–CC–p	68.17 ± 8.38	0.9698	0.274	0.83 ± 0.02	0.9932	0.022
Fe_3_O_4_–(CC–p)_2_	97.66 ± 9.07	0.9747	0.149	0.86 ± 0.02	0.9971	0.017
Fe_3_O_4_–CC–DETA	73.10 ± 6.62	0.9639	0.307	0.82 ± 0.02	0.9991	0.019
Fe_3_O_4_–(CC–DETA)_2_	132.45 ± 6.70	0.9682	0.172	0.77 ± 0.03	0.9995	0.019

**Table 3:** The description of the drug-release models applied for the fitting of the experimental data.

Drug Release Model	Linear Representation	Parameters
Zero-order model	$F_{t}=F_{0}+k_{0}t$	*F*_0_ and *F_t_*—the initial and the cumulative amount of the drug released at time *t*, respectively (mg) *k*_0_—the zero-order release constant (mg h^−1^) *k_H–C_*—the Hixson-Crowell release constant (mg^1/3^ h^−1^)
Hixson–Crowell model	$\sqrt[{3}]{F_{0}}-\sqrt[{3}]{F_{t}}=-k_{H-C}t$	
First-order model	$\log\left(100-Q_{t}\right)=-k_{1}t$	*Q_t_*—the cumulative percentage of the drug releases at time *t* (%) *k*_1_—the first-order release constant (% h^−1^) *k_H_*—the Higuchi release constant (% h^−1/2^) *n*—the Korsmeyer–Peppas exponent of release (–) *k_K–P_*—the Korsmeyer–Peppas release constant (% h^−1^)
Higuchi model	$Q_{t}=k_{H}\sqrt{t}$	
Korsmeyer–Peppas model	$\log Q_{t}=n\log t+\log k_{K-P}$	

**Table 4:** The parameters of the biocompounds’ releases from the materials functionalized with triazine dendrons of generation G2 (Fe_3_O_4_–(CC–p)_2_ and Fe_3_O_4_–(CC–DETA)_2_) in pH 2.0 and pH 7.4, calculated for the first-order, the Higuchi, and the Korsmeyer–Peppas release models.

	Adsorbent	Higuchi Model		Korsemeyer-Peppas Model		
		k_H_ (% h^−1/2^)	R^2^	*n*	k_K__–P_ (% h^−1^)	R^2^
**Folic Acid**						
pH 2.0	Fe_3_O_4_–(CC–p)_2_	2.7 ± 0.5	0.8561	0.06	62.7 ± 0.6	0.9676
	Fe_3_O_4_–(CC–DETA)_2_	2.4 ± 0.4	0.8435	0.05	71.4 ± 0.5	0.9717
pH 7.4	Fe_3_O_4_–(CC–p)_2_	3.5 ± 1.3	0.5944	0.14	37.1 ± 2.7	0.7633
	Fe_3_O_4_–(CC–DETA)_2_	4.4 ± 1.5	0.6341	0.15	41.0 ± 2.9	0.7994
**18β–Glycyrrhetinic Acid**						
pH 2.0	Fe_3_O_4_–(CC–p)_2_	4.1 ± 1.2	0.7087	0.10	55.6 ± 1.8	0.8967
	Fe_3_O_4_–(CC–DETA)_2_	3.0 ± 1.2	0.5353	0.07	67.0 ± 2.7	0.7295
pH 7.4	Fe_3_O_4_–(CC–p)_2_	6.2 ± 1.1	0.8603	0.27	23.3 ± 1.7	0.9273
	Fe_3_O_4_–(CC–DETA)_2_	5.8 ± 1.1	0.8388	0.22	28.5 ± 1.8	0.9192
**Vancomycin**						
pH 2.0	Fe_3_O_4_–(CC–p)_2_	3.8 ± 0.9	0.7603	0.08	70.0 ± 1.4	0.9302
	Fe_3_O_4_–(CC–DETA)_2_	1.7 ± 0.3	0.8205	0.03	86.0 ± 0.5	0.9581
pH 7.4	Fe_3_O_4_–(CC–p)_2_	4.6 ± 1.2	0.7395	0.13	47.5 ± 2.0	0.8922
	Fe_3_O_4_–(CC–DETA)_2_	2.7 ± 0.8	0.6832	0.07	60.8 ± 1.5	0.8664

## Data Availability

The data presented in this study are available on request from the corresponding author.

## Supplementary Materials

The following are available online at https://www.mdpi.com/article/10.3390/ijms222111353/s1.
