# Supplementary material for: Modification of Magnetite Nanoparticles with Triazine-Based Dendrons and Their Application as Drug-Transporting Systems
Source: Int J Mol Sci. 2021 Oct 21;22(21):11353. doi: 10.3390/ijms222111353 (PMC8583946; doi:10.3390/ijms222111353)
Supplement: Supplementary file 1 [file ijms-22-11353-s001.zip › ijms-1382908-supplementary.pdf]

Modification of Magnetite Nanoparticles with Triazine-Based Dendrons and Their Application as Drug-Transporting Systems

Mateusz Pawlaczyk * and Grzegorz Schroeder

Faculty of Chemistry, Adam Mickiewicz University in Poznań, Uniwersytetu Poznańskiego 8, 61-614 Poznań, Poland; schroede@amu.edu.pl

* Correspondence: mateusz.pawlaczyk@amu.edu.pl; Tel.: +48-618291797

Supplementary Information

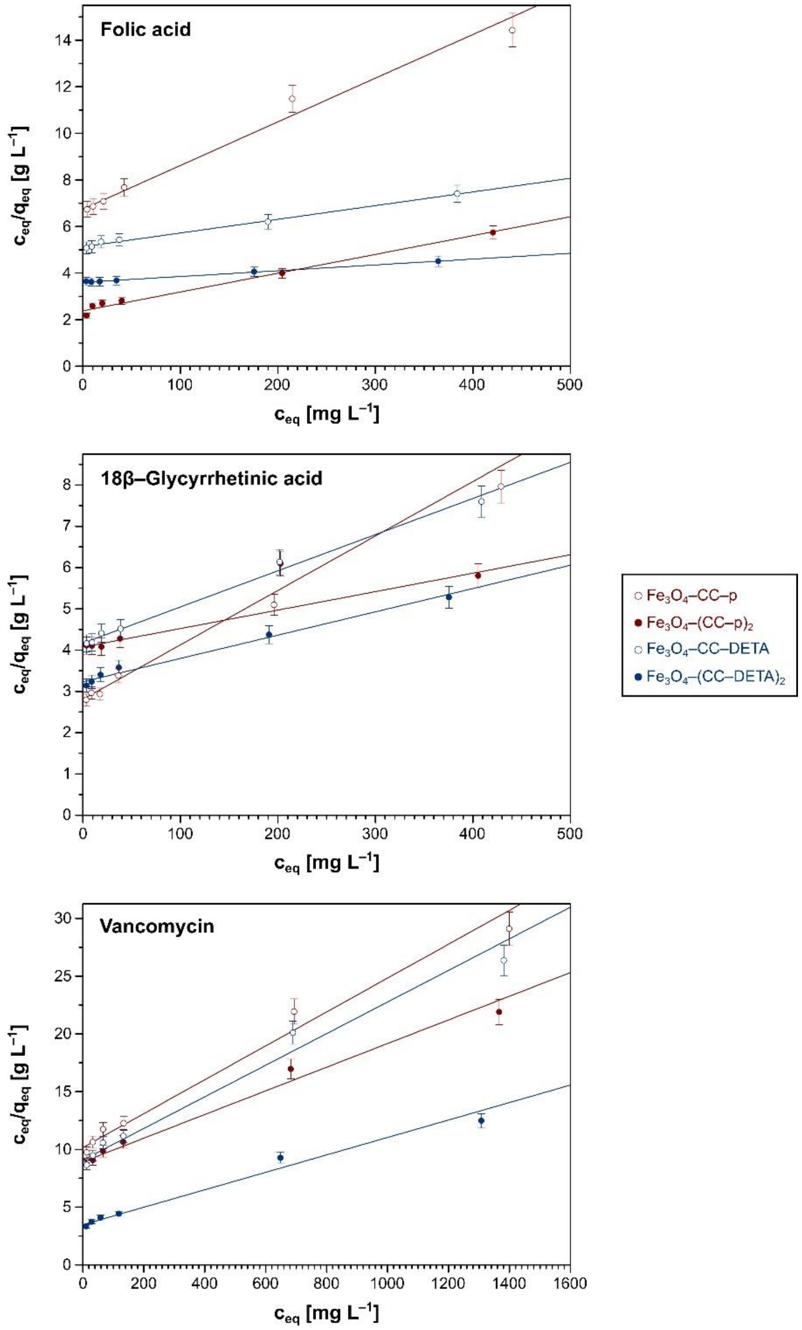

Figure S1. The fitting of the experimental data of isothermal studies to the Langmuir model.

Table S1. The parameters of the biocompound releases from the materials functionalized with triazine dendrons of generation G2 (Fe_3O_4–(CC–p)$_2$ and Fe_3O_4–(CC–DETA)$_2$) in pH 2.0 and pH 7.4, calculated for the zero-order, the first-order, and the Hixson–Crowell release models.

	Adsorbent	First-order model		Zero-order model		Hixson–Crowell model	
		$k_1 \cdot 10^2$ (% h^{-1})	R^2	$k_0 \cdot 10^3$ (mg h^{-1})	R^2	$k_{H-C} \cdot 10^2$ ($mg^{1/3}$ h^{-1})	R^2
**Folic acid**							
pH 2.0	Fe_3O_4–(CC–p)$_2$	0.48 ± 0.11	0.7840	0.41 ± 0.11	0.7025	0.07 ± 0.02	0.6763
	Fe_3O_4–(CC–DETA)$_2$	0.56 ± 0.13	0.7763	0.60 ± 0.18	0.6833	0.06 ± 0.02	0.6637
pH 7.4	Fe_3O_4–(CC–p)$_2$	0.31 ± 0.14	0.4766	0.62 ± 0.32	0.4188	0.12 ± 0.07	0.3702
	Fe_3O_4–(CC–DETA)$_2$	0.49 ± 0.19	0.5301	0.81 ± 0.40	0.4515	0.14 ± 0.08	0.3996
**18β–glycyrrhetinic acid**							
pH 2.0	Fe_3O_4–(CC–p)$_2$	0.61 ± 0.21	0.6055	0.79 ± 0.34	0.5192	0.11 ± 0.05	0.4883
	Fe_3O_4–(CC–DETA)$_2$	0.58 ± 0.26	0.4958	0.92 ± 0.53	0.3741	0.09 ± 0.05	0.3462
pH 7.4	Fe_3O_4–(CC–p)$_2$	0.56 ± 0.13	0.7819	1.26 ± 0.37	0.6983	0.26 ± 0.09	0.5966
	Fe_3O_4–(CC–DETA)$_2$	0.56 ± 0.14	0.7664	2.75 ± 0.85	0.6749	0.30 ± 0.11	0.5834
**Vancomycin**							
pH 2.0	Fe_3O_4–(CC–p)$_2$	1.05 ± 0.29	0.7209	1.82 ± 0.70	0.5759	0.13 ± 0.05	0.5509
	Fe_3O_4–(CC–DETA)$_2$	0.98 ± 0.20	0.8268	1.47 ± 0.47	0.6626	0.06 ± 0.02	0.6493
pH 7.4	Fe_3O_4–(CC–p)$_2$	0.58 ± 0.19	0.6563	1.66 ± 0.66	0.5588	0.17 ± 0.07	0.5102
	Fe_3O_4–(CC–DETA)$_2$	0.42 ± 0.16	0.5944	2.32 ± 1.02	0.5088	0.12 ± 0.06	0.4803
